# Physicians’ Vacations

**Published:** 1879-09

**Authors:** 


PHYSICIANS' VACATIONS.—One of our correspondents calls attention in the present number, to the subject of professional vacations. He seems to think a summer vacation or periodical recreation is as necessary for the physician as for the clergyman, the lawyer, the merchant, etc. And he intimates that the physician by resorting to some one of the fashionable watering places, would not only find recreation and recuperation, but could also keep up some profitable practice by professional attention to such of his home patrons as he might find at the same resorts. The reasoning of our correspondent might be good so far as pecuniary considerations are concerned, if the physician was so *unfortunate* as to have the principal part of his practice in such families as are able to take summer vacations and he can have them all spend their vacations in one place. But the truth is, that not one practitioner out of a thousand has such a practice; and not one family out of a thousand takes summer vacations. The fact is, that the great laboring classes constituting three-fourths of the human race, have no vacations. To them, the summer is more especially the season of active business, as well as the season of most sickness. Suppose the busy practitioners of medicine all take the advice of our correspondent, and follow some half dozen of their more wealthy patrons to the watering places and noted summer resorts, what is to become of the thousands left at home with their sick wives and babies? But even if all these were of no account, we should still doubt the correctness of the idea or principle that seems to underlie the modern practice of taking annual *vacations*. The prevalent idea appears to be that the merchant may confine himself to his counting-room from early morn to late in the evening; the lawyer to his office and the court-room; the clergyman to his study and church; the doctor to his patients; and the artisan to his shop; ten or eleven months of the year, regardless of daily rest, fresh air and needed exercise, all because they are going to have one or two months of vacation in which to recruit. If each in his individual avocation finds it difficult to keep his weary limbs and more weary head at its daily task until the set time for the vacation comes, he has only to smother his uncomfortable feelings by a little more of those narcotics and anæsthetics found in tobacco and the varieties of fermented and distilled drinks, or to go to his doctor for a blue pill and some bromide, meanwhile sustaining his courage by the remembrance that each day brings him nearer to his annual panacea, the vacation. Stript of its fashionable pretenses, all this modern noise about annual vacations for the maintenance of health, is founded upon the fallacious idea, that the latter is a commodity to be gathered in at some particular season, and stowed away in some part of the body, like a stock of merchandise or provisions, to be drawn upon for the rest of the year.

When all classes learn that health is a *condition* of a complicated and delicate organization, to be maintained by a daily balancing of the waste and supply, exercise and rest, and not a commodity to be stored up at one time and drawn upon at another, they will also learn to adjust their daily occupation more in accordance with physiological laws, and we shall hear much less about vacations. These remarks, of course do not apply to proper changes of climate for the purpose of aiding the removal of diseases or known predispositions to disease; nor to traveling in distant places or countries, either for business or pleasure; but simply to that very popular notion among all classes, that a vacation of a few weeks and a resort to some favorite place, is an ample remedy for the accumulated ills of all the rest of the year.

